# Survival in Korean Patients with Frontotemporal Dementia Syndrome: Association with Behavioral Features and Parkinsonism

**DOI:** 10.3390/jcm11082260

**Published:** 2022-04-18

**Authors:** Na-Yeon Jung, Kee Hyung Park, Sang Won Seo, Hee Jin Kim, Jee Hoon Roh, Jae-Hong Lee, Kyung Won Park, Jay C. Kwon, Jee Hyang Jeong, Soo Jin Yoon, Byeong C. Kim, Young Ho Park, SangYun Kim, Jae-Won Jang, Young Chul Youn, Dong Won Yang, Seong Hye Choi, Duk L. Na, Eun-Joo Kim

**Affiliations:** 1 Department of Neurology, Pusan National University Yangsan Hospital, Pusan National University School of Medicine, Yangsan 50612, Korea; i-me-mine@hanmail.net; 2 Research Institute for Convergence of Biomedical Science and Technology, Pusan National University Yangsan Hospital, Yangsan 50612, Korea; 3 Department of Neurology, College of Medicine, Gachon University Gil Hospital, Incheon 21565, Korea; khpark@gachon.ac.kr; 4 Department of Neurology, Samsung Medical Center, Sungkyunkwan University School of Medicine, Seoul 06351, Korea; sangwonseo@empas.com (S.W.S.); evekhj@gmail.com (H.J.K.); dukna@naver.com (D.L.N.); 5 Department of Biomedical Sciences, Korea University College of Medicine, Seoul 02841, Korea; alzheimer@naver.com; 6 Department of Physiology, Korea University College of Medicine, Seoul 02841, Korea; 7 Department of Neurology, Anam Hospital, Korea University College of Medicine, Seoul 02841, Korea; 8 Department of Neurology, Asan Medical Center, University of Ulsan College of Medicine, Seoul 05505, Korea; jhlee@amc.seoul.kr; 9 Department of Neurology, Dong-A Medical Center, Dong-A University College of Medicine, Busan 49201, Korea; neuropark@dau.ac.kr; 10 Department of Neurology, Changwon Fatima Hospital, Changwon 51394, Korea; chkwonj@hanmail.net; 11 Department of Neurology, Ewha Womans University College of Medicine, Seoul 07804, Korea; jjeong@ewha.ac.kr; 12 Department of Neurology, Eulji University Hospital, Eulji University School of Medicine, Daejeon 35233, Korea; trumind@eulji.ac.kr; 13 Department of Neurology, Chonnam National University Medical School, Gwangju 61469, Korea; byeong.kim7@gmail.com; 14 Department of Neurology, Seoul National University Bundang Hospital, Seoul National University College of Medicine, Seongnam 13620, Korea; kumimesy@snubh.org (Y.H.P.); neuroksy@snu.ac.kr (S.K.); 15 Department of Neurology, Kangwon National University Hospital, Kangwon National University School of Medicine, Chuncheon 24289, Korea; light26@kangwon.ac.kr; 16 Department of Neurology, Chung-Ang University Hospital, Chung-Ang University College of Medicine, Seoul 06973, Korea; neudoc@cau.ac.kr; 17 Department of Neurology, The Catholic University of Korea, Seoul St. Mary’s Hospital, Seoul 06591, Korea; neuroman@catholic.ac.kr; 18 Department of Neurology, Inha University School of Medicine, Incheon 22332, Korea; seonghye@inha.ac.kr; 19 Department of Neurology, Pusan National University Hospital, Pusan National University School of Medicine and Medical Research Institute, Busan 49241, Korea

**Keywords:** frontotemporal dementia, survival, abnormal behavior, parkinsonism

## Abstract

We investigated the survival time of each clinical syndrome of frontotemporal dementia (FTD) and the impacts of behavioral and motor features on survival of FTD. A total of 216 patients with FTD [82 behavioral variant FTD (bvFTD), 78 semantic variant primary progressive aphasia (svPPA), 43 non-fluent/agrammatic variant PPA (nfvPPA), 13 FTD-motor neuron disease (MND)] were enrolled from 16 centers across Korea. Behaviors and parkinsonism were assessed using the Frontal Behavioral Inventory and Unified Parkinson’s Disease Rating Scale Part III, respectively. The Kaplan–Meier method was used for the survival analysis and the Cox proportional hazards model was applied for analysis of the effect of behavioral and motor symptoms on survival, after controlling vascular risk factors and cancer. An overall median survival of FTD was 12.1 years. The survival time from onset was shortest for FTD-MND and longest for svPPA. The median survival time of patients with bvFTD was unavailable but likely comparable to that of patients with nfvPPA. In the bvFTD group, negative behavioral symptoms and akinetic rigidity were significantly associated with survival. In the nfvPPA group, the presence of dysarthria had a negative impact on survival. These findings provide useful information to clinicians planning for care.

## 1. Introduction

Frontotemporal dementia (FTD) has three distinct subtypes: behavioral variant FTD (bvFTD), semantic variant primary progressive aphasia (svPPA), and non-fluent/agrammatic variant PPA (nfvPPA). These often overlap in terms of their cognitive, behavioral, and motor symptoms. Motor neuron disease (MND) can develop in patients with FTD, or patients with MND may present with behavioral or language symptoms during the course of the disease (FTD-MND). 

The survival time of patients with neurodegenerative diseases is an important health issue for patients and families that are planning their medical care based on the natural history of the disease. Several survival analyses in FTD have been conducted over the last two decades. A recent meta-analysis showed that survival from symptom onset differed among FTD subtypes; the mean survival was 8 years for bvFTD and nfvPPA, the median survival was 12 years for svPPA and only 2–3 years for FTD-MND [1].

Despite an improved understanding of the clinical characteristics of FTD syndrome, their impact on survival is still unclear. Behavioral and neuropsychiatric symptoms are variably described in all FTD subtypes. Behavioral symptoms are associated with functional decline, increased mortality, and caregiver burden in patients with dementia and older people [2,3,4]. However, the relationship between behavioral disturbances and mortality in patients with FTD remains unclear. A recent study showed that a greater burden of behavioral symptoms predicted shorter survival in bvFTD; however, the authors used a tool not only to assess behavioral symptoms but also to incorporate cognition and activities of daily living (ADL) [5]. Additionally, parkinsonism is commonly observed in FTD syndrome [6]. About 20% of patients with bvFTD have parkinsonism at their first clinic visit [7]. In previous studies, the presence of parkinsonism had no significant effect on mortality [7,8]. However, the definition of parkinsonism used in the studies was an integrated form, which includes rigidity, resting tremor, and extrapyramidal gait. Bradykinesia or rigidity is more frequent than resting tremor in FTD [7,9]. Therefore, an approach is needed to determine how individual parkinsonian features are associated with survival in patients with FTD syndrome. 

In this study, we investigated (1) survival of each FTD subtype; (2) the effects of behavioral and individual parkinsonian features on survival in FTD using the behavioral scale specialized for FTD and the UPDRS motor scale; and (3) the effects of abnormal neurological findings. Only one Korean FTD survival study was conducted in a single tertiary center; thus, it might not be representative of the Korean FTD population [10]. In addition, most previous studies did not consider the effect of comorbid conditions such as hypertension, diabetes mellitus, heart disease, and cancer on survival. In this regard, our multicenter study overcame the limitations of previous studies by adjusting for vascular risk factors and cancer, which may influence mortality. 

## 2. Materials and Methods

### 2.1. Patients

A total of 216 patients with FTD (82 bvFTD, 78 svPPA, 43 nfvPPA, and 13 FTD-MND) were recruited from 16 centers participating in the Clinical Research Center for Dementia of South Korea (CREDOS)-FTD registry between January 2010 and February 2015. bvFTD was diagnosed based on the international consensus criteria for probable bvFTD [11]. The diagnosis of PPA was also made using the recommendations of Gorno-Tempini et al. in 2011 [12]. Patients with FTD-MND were defined as FTD patients with clinical and electrophysiological evidence of MND, regardless of the clinical subtype of FTD. 

All patients were evaluated by comprehensive interviews, neurological examinations, neuropsychological assessments, and neuroimaging. The dates of death until 31 December 2016 were recorded for all participants based on information from the National Health Insurance Service. Written informed consent was obtained from all patients and their caregivers. This study was approved by the institutional review board of all participating centers.

### 2.2. Behavioral Assessment

Behavioral symptoms were assessed using the Frontal Behavioral Inventory (FBI) [13,14]. The FBI was specifically developed to measure behavioral disturbances in FTD [13,14]. It is a 24-item caregiver questionnaire, half of which assesses deficit or negative behaviors, and the other half assesses disinhibited or positive behaviors. Negative behaviors include apathy, aspontaneity, indifference/emotional flatness, inflexibility, personal neglect, disorganization, inattention, loss of insight, logopenia, comprehension deficit, aphasia/verbal apraxia, and alien hand and/or apraxia. Positive behaviors include perseveration/obsession, irritability, excessive jocularity, impulsivity/poor judgement, hoarding, inappropriateness, restlessness/roaming, aggression, hyperorality, hypersexuality, utilization behavior, and incontinence. The FBI assesses behavior on a 4-point scale that incorporates severity and frequency (never = 0, mild or occasional = 1, moderate = 2, and severe or very frequent = 3). The FBI total score is the sum of all items, with a maximum score of 72. The subtotal scores of the 12 negative items (FBI-negative) and 12 positive items (FBI-positive) were based on the addition of items, with a maximum score of 36. 

### 2.3. Assessment of Parkinsonism and Neurological Examinations

Parkinsonism was evaluated using the Unified Parkinson’s Disease Rating Scale (UPDRS) Part III, and the presence of parkinsonism was defined if any of the following conditions were met: (1) 2 or more UPDRS ratings of 1; (2) 1 UPDRS rating ≥ 2; or (3) a UPDRS resting tremor rating ≥ 1 [15]. To analyze the effect of individual parkinsonian features on survival, UPDRS motor scores were divided into five components: (i) speech/facial expression, (ii) tremor, (iii) rigidity, (iv) bradykinesia, and (v) gait/posture [16]. Cranial nerve, motor, sensory, and reflex examinations are typically normal in FTD [17]. However, there are neurological deficits such as dysarthria in nfvPPA patients; in addition, motor weakness is present in MND patients. Therefore, neurological examinations were performed to check for dysarthria, extraocular muscle (EOM) limitation, facial palsy, motor weakness, sensory loss, pathological reflex, or abnormal deep tendon reflex. Motor weakness was defined as weakness in the upper and lower limbs. 

### 2.4. Statistics

For descriptive statistics, the χ^2^ test or Fisher’s exact test and analysis of variance followed by Bonferroni’s post hoc analysis were used to compare the subtypes of FTD.

The Kaplan–Meier method was used for the survival analysis by diagnostic group. Survival curves were compared using log-rank tests. To examine the effect of behavioral symptoms, parkinsonism, and neurological deficits on survival, we used the Cox proportional hazards model with backward stepwise regression to eliminate non-significant variables, adjusting for age of onset, sex, years of education, MMSE, vascular risk factors (hypertension, diabetes, hyperlipidemia, heart disease, cerebrovascular attack), and cancer. Since too few events of neurological deficits lead to low predictive accuracy, variables detected in >10% of the total patients were entered into the Cox proportional hazards model [18].

## 3. Results

### 3.1. Demographic, Neurological, Behavioral, and Parkinsonian Features

Of the 216 patients, 67 (31.0%) died during the study period. Age at onset, age at diagnosis, age at assessment, sex, education level, and vascular risk factors, except for diabetes, were not significantly different among the four clinical subtypes. The frequency of diabetes was higher in the bvFTD group than in the svPPA and FTD-MND groups (Table 1). 

The total FBI score was higher in the bvFTD group than in the svPPA and nfvPPA groups. The FBI total score was lower in the nfvPPA group than in the other groups. In the comparison using subtotal scores, the bvFTD and svPPA groups showed more severe negative and positive behaviors than the nfvPPA group. The bvFTD group showed more severe positive behaviors than the svPPA group. The FTD-MND group exhibited similar degrees of abnormal behavior to those in the bvFTD group (Table 2). 

The frequency of parkinsonism was the most common in FTD-MND, followed by nfvPPA, bvFTD, and svPPA (Table 2). However, there was no significant difference in the total UPDRS scores among the four clinical subtypes. The UPDRS score for bradykinesia was significantly higher in the nfvPPA group than in the svPPA group. The bvFTD, nfvPPA, and FTD-MND groups showed significantly higher scores for speech/facial expression than the svPPA group. There were no significant differences in tremor, rigidity, and gait/posture among the groups. 

Neurological examinations revealed that dysarthria and motor weakness were more frequent in the FTD-MND group than in the other groups. The nfvPPA group had a higher frequency of dysarthria than the bvFTD and svPPA groups. The frequencies of EOM limitation, facial palsy, sensory loss, deep tendon reflex (DTR), and Babinski/Chaddock signs did not differ between the groups (Table 1).

### 3.2. Survival Times and Its Associated Factors

**Figure 1:**
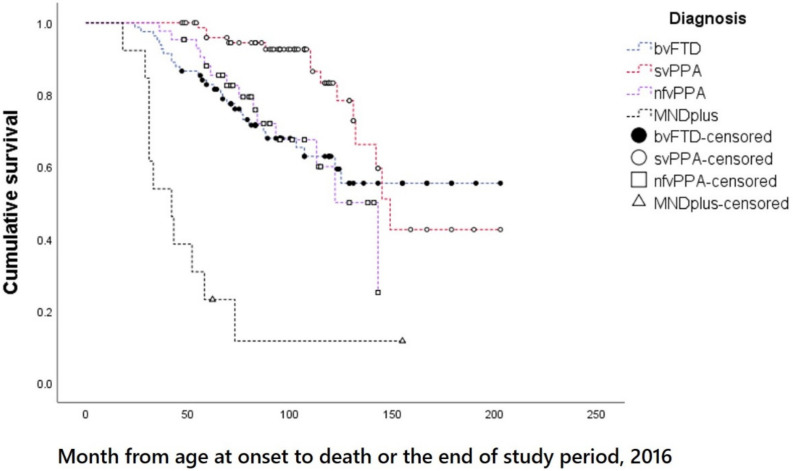
Kaplan-Meier survival plots for the 4 subtypes of FTD. Abbreviations: bvFTD, behavioral variant frontotemporal dementia; FTD-MND, frontotemporal dementia with motor neuron disease; nfvPPA, non-fluent/agrammatic variant primary progressive aphasia; svPPA, semantic variant primary progressive aphasia.

The mean observational period was 4.4 years. The overall median survival in the FTD cohort from the onset of the first symptom was 12.1 years. Among the FTD subtypes, the median survival time from onset was shortest for FTD-MND (3.5 y) and longest for svPPA (12.4 y). The median survival time of patients with bvFTD could not be obtained because the cumulative survival of bvFTD patients was over 50% during the observational period (Table 1, Figure 1). The overall median survival time in FTD from diagnosis was 8.8 years (range: 7.3 to 10.2 y). The survival time from diagnosis was shortest for FTD-MND (1.3 y) (Table 1).

Survival times from onset significantly differed between subtypes (log-rank [Mantel–Cox] χ^2^ = 56.8, df = 3, *p <* 0.001; pairwise comparisons revealed significant differences for bvFTD vs. svPPA: χ^2^ = 6.0, *p =* 0.014; bvFTD *vs.* FTD-MND: χ^2^ = 29.2, *p <* 0.001; svPPA vs. nfvPPA: χ^2^ = 8.8, *p =* 0.003; svPPA vs. FTD-MND: χ^2^ = 49.0, *p <* 0.001; and nfvPPA vs. FTD-MND: χ^2^ = 22.5, *p <* 0.001). There was no significant difference in survival time from onset between bvFTD and nfvPPA (χ^2^ = 0.002, *p =* 0.963). Similarly, survival times from diagnosis significantly differed between subtypes (log-rank [Mantel–Cox] χ^2^ = 56.8, df = 3, *p <* 0.001; pairwise comparisons revealed significant differences for bvFTD vs. svPPA: χ^2^ = 8.2, *p =* 0.004; bvFTD vs. FTD-MND: χ^2^ = 31.3, *p <* 0.001; svPPA vs. nfvPPA: χ^2^ = 6.0, *p =* 0.014; svPPA vs. FTD-MND: χ^2^ = 64.5, < 0.001; and nfvPPA vs. FTD-MND: χ^2^ = 30.1, *p <* 0.001). There was no significant difference in the survival time from diagnosis between bvFTD and nfvPPA (χ^2^ = 0.131, *p =* 0.717).

In the Cox proportional hazards model, a higher FBI total score was associated with shorter survival in all patients (HR = 1.020, *p =* 0.008) and the bvFTD group (HR = 1.037, *p =* 0.012). Among the individual items, apathy, aspontaneity, inattention, logopenia, aphsia/verbal apraxia, and hyperorality were significantly associated with survival, but others did not show any associations. Since the majority of significant FBI individual items were negative behaviors (apathy, aspontaneity, inattention, logopenia, aphasia/verbal apraxia), we analyzed the effect of FBI negative and positive subscores on survival. FBI negative scores were significantly associated with survival in all patients and the bvFTD group, whereas FBI positive scores were not associated with survival in all patients and any group (Table 3). 

Higher UPDRS scores of speech/facial expression and bradykinesia were associated with shorter survival in all patients. UPDRS scores of speech/facial expression, rigidity, bradykinesia, and gait/posture were associated with survival in the bvFTD group (Table 3). There was no significant association between parkinsonian features and survival in the svPPA, nfvPPA, and FTD-MND groups.

Dysarthria and decreased DTR that were detected in >10% of the total patients were entered into the Cox proportional hazard model to determine their association with survival [18]. In the nfvPPA group, the presence of dysarthria was associated with survival. 

## 4. Discussion

In our multicenter study evaluating the survival of patients with FTD after adjusting for vascular risk factors and cancers, the three major findings were as follows. First, negative behaviors were associated with survival in the total FTD and bvFTD subgroups. Second, bradykinesia and rigidity were associated with survival in the bvFTD group. Third, dysarthria was found to be associated with survival in the nfvPPA group. 

Among the FTD subtypes, the svPPA group had a longer survival time from onset and diagnosis than the other subtypes, whereas the FTD-MND group had a shorter survival time than the other subtypes. There was no difference in the median survival times from onset and diagnosis between the bvFTD and nfvPPA groups. These results are generally consistent with those of previous studies [5,19,20,21]. 

Negative behaviors were associated with shorter survival in patients with bvFTD. Among the 12 negative behaviors, apathy (HR = 1.619), aspontaneity (HR = 1.540), indifference/emotional flatness (HR = 1.547), comprehension deficit (HR = 1.668), and aphasia/verbal apraxia (HR = 1.606) were associated with survival in patients with bvFTD. Apathy is a representative negative behavior. In cognitively impaired older adults, apathy is strongly associated with mortality [4]. Apathy can influence multiple directions in neurodegenerative diseases. When patients with Alzheimer’s disease or Parkinson’s disease have greater apathy, their quality of life significantly declines [22,23]. In addition, apathy is associated with slow gait, frailty [24], poor nutrition [25], poor medication adherence, and cardiovascular diseases [26] which also negatively impact mortality [27,28,29,30]. The relationship between negative behaviors and health problems has been frequently investigated in schizophrenia [31]. Patients with schizophrenia die earlier than the general population [32]. High cardiovascular risk associated with negative symptoms [31] has also been suggested as an explanation for the early mortality of patients with schizophrenia [33]. Therefore, a high risk of poor lifestyles and cardiovascular diseases associated with negative behaviors may be involved in the shorter survival of patients with bvFTD. 

In terms of biology, it has been hypothesized that positive behaviors occur as a result of deficient inhibitory circuits (GABA), while negative behaviors arise following a loss of excitatory circuits (glutamate) in psychiatric disorders [34]. Loss of glutamatergic pyramidal neurons is a critical neuropathological involvement in FTD [35,36]; thus, targeting glutamatergic transmission might be a potential therapeutic approach [36]. 

Speech/facial expression, rigidity, bradykinesia, and gait/posture problems were associated with survival in the bvFTD group. The effect of parkinsonism on survival has been well studied in progressive supranuclear palsy (PSP) and corticobasal syndrome (CBS). Early falling was a predictive factor of poor survival in patients with PSP [37]. Extrapyramidal symptoms also predicted shorter survival in CBS patients [38]. In Parkinson’s disease (PD), worse parkinsonian impairment is a prognostic factor associated with mortality [39]. However, few studies have investigated the effect of parkinsonism on survival in patients with FTD syndromes. Previous studies have shown that parkinsonism does not significantly affect survival in FTD [7,8]. The studies used the existence or nonexistence of parkinsonism itself, rather than individual parkinsonism scales, in their survival analysis. In addition, parkinsonism in previous studies was less frequent (3–20%) than in our study (38–61%, except for FTD-MND) [19]. The reason for the different frequencies might be that we included mild parkinsonism. Similarly to our results, in PD patients, cardinal motor features, except tremor, were associated with mortality [40]. In particular, predominant bradykinesia or postural instability gait disorder (PIGD) phenotypes are prognostic factors associated with mortality in PD patients [39,40,41]. A possible mechanism of the underlying prognosis for motor subtypes may be explained by the extent of neuropathology and neuronal injury. The akinetic/rigid or non-tremor dominant PD patients had more extensive deposits of Lewy bodies than the tremor-dominant phenotype patients [40,42]. Therefore, widespread neurodegeneration may be the basis for the adverse effects of non-tremor-dominant parkinsonism on survival. Another mechanism suggested that reduced physical activity due to parkinsonism may be related to cardiovascular disease. PD increases the risk of all-cause mortality in the general population [43]; pneumonia and cardiovascular disease, and injury caused by falling are the main causes of mortality among patients with PD [43]. In addition, it cannot be excluded that bvFTD-PSP or bvFTD-CBD, which presents as bvFTD and later develops typical motor symptoms of PSP or CBD, finally turning out to be FTLD-tau, PSP, or CBD pathology was enrolled in our study population [44,45].

Interestingly, of the neurological deficits, dysarthria was associated with survival in the nfvPPA group. Dysarthria is a strong predictor of dysphagia, a risk for aspiration [46]. Aspiration pneumonia is associated with mortality in the late stage of neurodegenerative diseases. Dysarthria is a cardinal sign of classical PSP-Richardson syndrome (PSP-RS) or CBS linked to FTLD-tau pathology [47]. NfvPPA often develops into PSP-RS or CBS, which leads to shorter survival times than other FTD syndromes [20,48,49]. The early presence of dysarthria in patients with nfvPPA has been shown to have underlying PSP pathology [50]. A previous study also reported that nfvPPA patients with dysarthria showed more atrophy of the left primary motor cortex and caudate than those without dysarthria [51]. Thus, the presence of dysarthria in nfvPPA, indicating underlying PSP pathology or greater neurodegeneration, might contribute to shorter survival in patients with nfvPPA. 

We acknowledge that this study was based on clinical diagnosis and not on autopsy-proven cases, which is a limitation of our study. Future studies investigating the associations between underlying pathologies and survival in FTD syndromes are needed. However, the strength of our study lies in our survival analyses in which we used detailed behavioral and motor scales for each FTD subtype while considering cardiovascular risk factors and other comorbidities.

## 5. Conclusions

The overall Korean FTD median survival from onset was 12.1 years. FTD-MND showed the shortest median survival (3.5 years), whereas svPPA had the longest median survival (12.4 years). Negative behavioral symptoms were associated with shorter survival in patients with bvFTD, but positive symptoms were not associated with survival in any group. In bvFTD, the degree of speech/facial expression, rigidity, bradykinesia, and gait/posture were associated with poor survival. Finally, dysarthria prognosticated shorter survival in the nfvPPA group.

## Figures and Tables

**Table 1:** Demographic characteristics and median survival of the study population.

	Total	bvFTD	svPPA	nfvPPA	FTD-MND	*p*
Subjects/death, *n*	216/67	82/28	78/14	43/14	13/11	
Male (%)	109 (50.5)	44 (53.7)	33 (42.3)	25 (58.1)	7 (53.8)	0.323
Education (y)	9.7 ± 5.1	9.8 ± 5.1	9.6 ± 4.9	10.4 ± 5.7	7.9 ± 4.3	0.487
Age at onset (y)	62.6 ± 9.3	62.1 ± 10.6	62.5 ± 7.4	64.7 ± 9.2	60.5 ± 10.5	0.371
Age at diagnosis(y)	65.6 ± 8.9	65.1 ± 10.2	65.8 ± 7.4	67.1 ± 8.8	62.9 ± 10.0	0.447
Age at assessment (y)	65.9 ± 8.9	65.3 ± 10.2	66.3 ± 7.3	67.3 ± 8.8	63.1 ± 9.7	0.400
Onset-assessment interval (months)	39.2 ± 26.8	39.5 ± 28.6	44.9 ± 27.9	31.1 ± 20.6 ^c^	29.2 ± 18.1	0.024
MMSE	19.0 (7.7)	19.4 (6.8)	18.3 (8.8)	20.6 (7.3)	14.9 (6.7)	0.109
CDR-SB	5.7 (4.3)	6.9 (4.2)	5.4 (4.4)	3.5 (3.2) ^a,b^	7.8 (4.8)	<0.001
FTD CDR-SB	11.2 (6.4)	9.5 (5.2)	8.0 (5.5)	5.7 (3.9) ^a,b^	11.2 (6.4)	<0.001
Diabetes, *n* (%)	37 (17.4)	21 (26.6) ^d,g^	10 (12.8)	6 (14.0)	0 (0)	0.031
Hypertension	79 (36.9)	33 (41.3)	22 (28.2)	17 (39.5)	7 (53.8)	0.178
Hyperlipidemia	30 (14.0)	14 (17.5)	9 (11.5)	5 (11.6)	2 (15.4)	0.699
Heart disease	20 (9.4)	11 (13.9)	7 (9.0)	2 (4.7)	0 (0)	0.221
Stroke	6 (2.8)	3 (3.8)	1 (1.3)	1 (2.3)	1 (7.7)	0.549
Cancer	14 (6.6)	4 (5.1)	3 (3.8)	6 (14.0)	1 (7.7)	0.166
Neurological Examination (*n* = 145)						
Dysarthria	16 (11)	3 (5.3)	0 (0)	7 (22.6) ^a,c^	6 (66.7) ^b,f,g^	<0.001
EOM limitation	3 (2.1)	2 (3.5)	0 (0)	1 (3.2)	0 (0)	0.546
Facial palsy	1 (0.7)	1 (1.8)	0 (0)	0 (0)	0 (0)	1.000
Motor weakness	8 (5.5)	2 (3.5)	1 (2.1)	0 (0)	5 (55.6) ^b,f,g^	<0.001
Sensory loss	1 (0.7)	1 (1.8)	0 (0)	0 (0)	0 (0)	1.000
Increased DTR	9 (6.2)	5 (8.8)	1 (2.1)	1 (3.2)	2 (22.2)	0.090
Decreased DTR	23 (15.9)	9 (15.8)	6 (12.5)	4 (12.9)	4 (44.4)	0.148
Babinski/Chaddock	13 (9.0)	6 (10.5)	5 (10.4)	1 (3.2)	1 (11.1)	0.588
Median survival from onset to death (y, 95% CI)	12.1	NA	12.4 ± 0.4 ^d,e,f^ (11.6–13.3)	10.2 ± 0.9 (8.4–11.9)	3.5 ± 0.6 ^b,g^ (2.3–4.7)	<0.001
Median survival from diagnosis to death (y, 95% CI)	8.8 ± 0.7 (7.3–10.2)	NA	9.0 ± 0.8 ^d,e,f^ (7.4–10.6)	8.8 ± 0 (NA)	1.3 ± 0.3 ^b,g^ (0.7–1.9)	<0.001

We used the χ_2_ test or Fisher’s exact test to compare dichotomous variables and analysis of variance followed by Bonferroni’s post hoc analysis to compare continuous variables among the FTD subtypes. Data are expressed as the mean ± standard deviation or number of subjects (%). The Kaplan–Meier method was used for survival analysis in the FTD subtype group. Survival was compared with the log-rank test; results are presented as median survival ± standard error. ^a^ bvFTD vs. nfvPPA <0.05; ^b^ nfvPPA vs. FTD-MND <0.05; ^c^ svPPA vs. nfvPPA <0.05; ^d^ bvFTD vs. svPPA <0.05; ^e^ svPPA vs. nfvPPA <0.05; ^f^ svPPA vs. FTD-MND <0.05; ^g^ bvFTD vs. FTD-MND <0.05. Abbreviations: bvFTD, behavioral variant frontotemporal dementia; CDR-SB, clinical dementia rating sum of boxes; DTR, deep tendon reflex; EOM, extraocular movement; FTD CDR-SB, frontotemporal dementia clinical dementia rating sum of boxes; FTD-MND, frontotemporal dementia with motor neuron disease; MMSE, Mini-Mental State Examination; nfvPPA, non-fluent/agrammatic variant primary progressive aphasia; svPPA, semantic variant primary progressive aphasia; y, years.

**Table 2:** Behavioral and parkinsonian features.

	Total (*n* = 216)	bvFTD (*n* = 82)	svPPA (*n* = 78)	nfvPPA (*n* = 43)	FTD-MND (*n* = 13)	*p*
FBI total ^†^	26.6 ± 15.2	32.5 ± 13.1 ^a,d^	25.9 ± 16.1 ^b^	16.6 ± 11.9	28.7 ± 14.5 ^e^	<0.001
FBI_negative	17.8 ± 9.2	20.5 ± 8.4 ^d^	17.5 ± 9.6 ^b^	12.5 ± 8.0	20.1 ±9.4 ^e^	<0.001
FBI_positive	8.9 ± 7.5	12.0 ± 7.3 ^a,d^	8.4 ± 7.9 ^b^	4.1 ± 4.7	8.6 ± 5.7	<0.001
Presence of Parkisonism, *n* (%) *	104 (51.5)	41 (53.9)	28 (38.4) ^c^	25 (61.0)	10 (83.3)	0.009
UPDRS total score	7.0 (11.6)	8.4 (12.5)	4.1 (10.1)	9.3 (12.8)	8.2 (6.1)	0.055
UPDRS Speech/facial	1.0 ± 1.5	1.1 ± 1.5	0.4 ± 1.2 ^a,b,c^	1.5 ± 1.6	1.8 ± 1.9	<0.001
UPDRS UPDRS Tremor	0.5 ± 1.3	0.7 ± 1.8	0.2 ± 0.6	0.5 ± 0.9	0.9 ± 2.1	0.102
UPDRS Rigidity	1.1 ± 2.8	1.6 ± 3.3	0.7 ± 2.5	1.2 ± 2.4	0.3 ± 1.2	0.221
UPDRS bradykinesia	3.4 ± 5.6	3.7 ± 5.6	2.0 ± 4.4	5.0 ± 7.1 ^b^	4.9 ± 4.5	0.026
UPDRS Gait/posture	1.0 ± 2.3	1.2 ± 2.4	0.7 ± 2.3	1.2 ± 2.5	0.3 ± 0.6	0.386

We used the χ^2^ test or Fisher’s exact test to compare dichotomous variables and analysis of variance followed by Bonferroni’s post hoc analysis to compare continuous variables among the FTD subtypes. Continuous variables are expressed as mean ± standard deviation. ^a^ bvFTD vs. svPPA < 0.05; ^b^ svPPA vs. nfvPPA < 0.05; ^c^ svPPA vs. FTD-MND < 0.05; ^d^ bvFTD vs. nfvPPA < 0.05; ^e^ nfvPPA vs. FTD-MND < 0.05. ^†^ Missing data of FBI exists in 5 patients (4 bvFTD and 1 nfvPPA patients). * Missing data of parkinsonism exists in 14 patients (6 bvFTD, 5 svPPA, 2 nfvPPA, and 1 FTD-MND patients). Abbreviations: bvFTD, behavioral variant frontotemporal dementia; FBI, Frontal Behavioral Inventory; FTD-MND, frontotemporal dementia with motor neuron disease; nfvPPA, non-fluent/agrammatic variant primary progressive aphasia, svPPA, semantic variant primary progressive aphasia; UPDRS, Unified Parkinson’s Disease Rating Scale.

**Table 3:** Effect of factors on survival from onset.

	Total		bvFTD		svPPA		nfvPPA		FTD-MND	
	*p*	HR (95% CI)	*p*	HR (95% CI)	*p*	HR (95% CI)	*p*	HR (95% CI)	*p*	HR (95% CI)
FBI total	0.008	1.020 (1.005–1.035)	0.012	1.037 (1.008–1.067)	n.s	n.s	n.s	n.s	n.s	n.s
FBI_negative	0.004	1.041 (1.013–1.070)	0.003	1.110 (1.037–1.187)	n.s	n.s	n.s	n.s	n.s	n.s
FBI_positive	n.s	n.s	n.s	n.s	n.s	n.s	n.s	n.s	n.s	n.s
UPDRS speech/facial	0.024	1.161 (1.020–1.322)	0.032	1.324 (1.025–1.711)	n.s	n.s	n.s	n.s	n.s	n.s
UPDRS tremor	n.s	n.s	n.s	n.s	n.s	n.s	n.s	n.s	n.s	n.s
UPDRS rigidity	n.s	n.s	0.004	1.171 (1.053–1.302)	n.s	n.s	n.s	n.s	n.s	n.s
UPDRS bradykinesia	0.022	1.041 (1.006–1.077)	<0.001	1.140 (1.064–1.222)	n.s	n.s	n.s	n.s	n.s	n.s
UPDRS gait/posture	n.s	n.s	<0.001	1.245 (1.102–1.407)	n.s	n.s	n.s	n.s	n.s	n.s
Dysarthria	<0.001	5.413 (2.788–10.507)	n.s	n.s	NA	NA	0.030	7.593 (1.221–47.226)	NA	NA
Decreased DTR	n.s	n.s	n.s	n.s	n.s	n.s	n.s	n.s	n.s	n.s

The Cox proportional hazards model was used to adjust for onset age, sex, education, MMSE, hypertension, diabetes, hyperlipidemia, heart disease, cerebrovascular attack, and cancer. Abbreviations: bvFTD, behavioral variant frontotemporal dementia; CI, confidence interval; DTR, deep tendon reflex; FBI, Frontal Behavioral Inventory; FTD-MND, frontotemporal dementia with motor neuron disease; HR, hazard ratio; NA, not available; nfvPPA, non-fluent/agrammatic variant primary progressive aphasia; n.s, not significant; svPPA, semantic variant primary progressive aphasia; UPDRS, Unified Parkinson’s Disease Rating Scale.

## Data Availability

The data presented in this study are available in Supplementary Materials.

## Supplementary Materials

The following are available online at https://www.mdpi.com/article/10.3390/jcm11082260/s1, Supplemental file: Data.
